# Right Heart Clot in Transit: A Case Report of Acute Submassive Pulmonary Embolism

**DOI:** 10.7759/cureus.84909

**Published:** 2025-05-27

**Authors:** Kubiat E Udoh, Kuseme E Udoh, Joy Efik, Andikan E Udoh

**Affiliations:** 1 Internal Medicine, Baton Rouge General, Baton Rouge, USA; 2 Internal Medicine, Sumy State University, Sumy, UKR

**Keywords:** anticoagulation, clot in transit, pulmonary embolism, right heart clot, submassive pulmonary embolism

## Abstract

Pulmonary embolism (PE) is classified into massive, submassive, and low-risk categories, with severity often assessed using tools such as the Pulmonary Embolism Severity Index (PESI) and simplified PESI (sPESI) to determine inpatient versus outpatient management. This report discusses an 84-year-old male with a history of prostate cancer (not on chemotherapy), hypertension, type 2 diabetes mellitus, and hyperlipidemia, who developed bilateral PE and a right heart thrombus in transit after a syncopal episode during hospitalization for a viral illness. A right heart thrombus in transit is a mobile clot within the right heart chambers or vena cava, posing a high risk for further embolization. These thrombi are often visualized on echocardiography and are considered a medical emergency. Although the patient remained hemodynamically stable, evidence of right ventricular dysfunction confirmed by laboratory markers (e.g., elevated troponin and BNP levels) and imaging studies was consistent with the diagnosis of submassive PE.

Management of right heart thrombi in transit remains controversial due to the lack of randomized controlled trials. Treatment options include systemic anticoagulation, systemic or catheter-directed thrombolysis, catheter-based embolectomy, and surgical thrombectomy. In elderly patients with multiple comorbidities, selecting the optimal approach requires multidisciplinary input. This case highlights the critical need for multidisciplinary evaluation and the complexity of managing right heart thrombi and the importance of individualized treatment strategies in high-risk patients.

## Introduction

A right heart thrombus (RHT) in transit is a rare but clinically significant finding. It represents a mobile clot within the right atrium or ventricle, sometimes extending into the superior or inferior vena cava. Its presence indicates an impending or ongoing pulmonary embolism and is considered a medical emergency due to the high risk of sudden and potentially fatal embolization of the pulmonary arteries [1].

Although pulmonary embolism (PE) is a relatively common cardiopulmonary emergency encountered in clinical practice [2], the presence of a right heart thrombus alters the clinical picture and management urgency [2,3]. PE may present acutely or subacutely, with symptoms ranging from dyspnea, chest pain, palpitations, and syncope to hemodynamic compromise or obstructive shock in severe cases, evidenced by hypotension and signs of end-organ hypoperfusion (e.g., oliguria, altered mental status) [2,3]. Submassive pulmonary embolism is associated with RV strain, which is classified by the presence of cardiac biomarkers and imaging showing evidence of RV dysfunction in the absence of hemodynamic compromise [3].

Risk stratification scores such as the Pulmonary Embolism Severity Index (PESI) and its simplified version (sPESI) are widely used once PE is diagnosed. These scores incorporate clinical variables including age, comorbidities, vital signs, and oxygenation to predict short-term mortality and guide decisions regarding treatment intensity and level of care [2,3].

In this report, we describe an 84-year-old male with a history of prostate cancer (not on chemotherapy) who experienced a syncopal episode and was subsequently found to have large bilateral pulmonary emboli on imaging, complicated by a right heart thrombus in transit. His clinical presentation followed an indolent course characterized by gradual progression, minimal cardiorespiratory symptoms, and the absence of hemodynamic instability, which is atypical in cases involving right heart thrombi. This highlights the diagnostic challenge, such presentations pose and underscores the importance of maintaining a high index of suspicion and conducting a thorough evaluation, even when symptoms appear mild or non-specific.

## Case presentation

**Figure 1:**
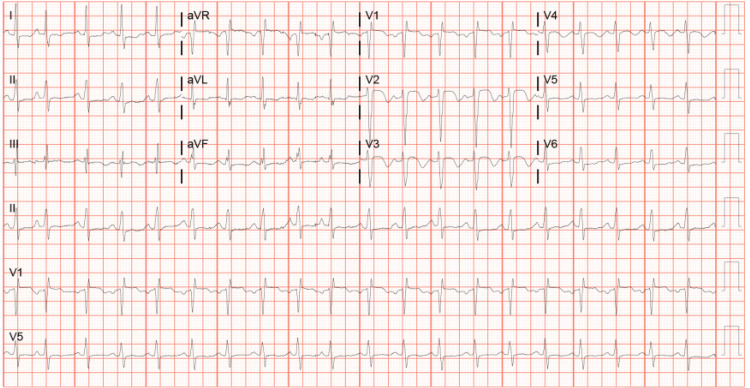
EKG showing sinus tachycardia and presence of a right bundle branch block. EKG: electrocardiogram

An 84-year-old male with a history of prostate cancer (not on chemotherapy), hypertension, non-insulin-dependent diabetes mellitus, and hyperlipidemia presented to the hospital with a one-week history of exertional shortness of breath, accompanied by generalized malaise. On arrival at the hospital, he initially required supplemental oxygen, and his vital signs were as follows: blood pressure 113/68 mmHg, heart rate 128 beats per minute, respiration rate 30 breaths per minute, and oxygen saturation 94% on 2 L of oxygen. Table 1 illustrates the pertinent laboratory results as follows: troponin 373.4 pg/mL, brain natriuretic peptide (BNP) 934.8 pg/mL, creatinine 2.19 mg/dL, and he also tested positive for influenza B. Figure 1 illustrates the electrocardiogram (EKG) which showed sinus tachycardia and presence of a right bundle branch block. His cardiac biomarkers were notably elevated, but the patient denied chest pain, palpitations, or dyspnea. Intravenous fluids were initiated, and he was treated supportively for a viral syndrome.

**Table 1:** Laboratory results highlighting the cardiac biomarkers and renal function on admission. BUN: blood urea nitrogen; BNP: brain natriuretic peptide

Chemistry	Laboratory results	Reference ranges
BUN	24 mg/dL	7-18 mg/dL
Creatinine	2.19 mg/dL	0.7-1.3 mg/dL
Troponin	373.4 pg/mL	0-58.9 pg/mL
BNP	934.8 pg/mL	0-99.9 pg/mL

**Figure 2:**
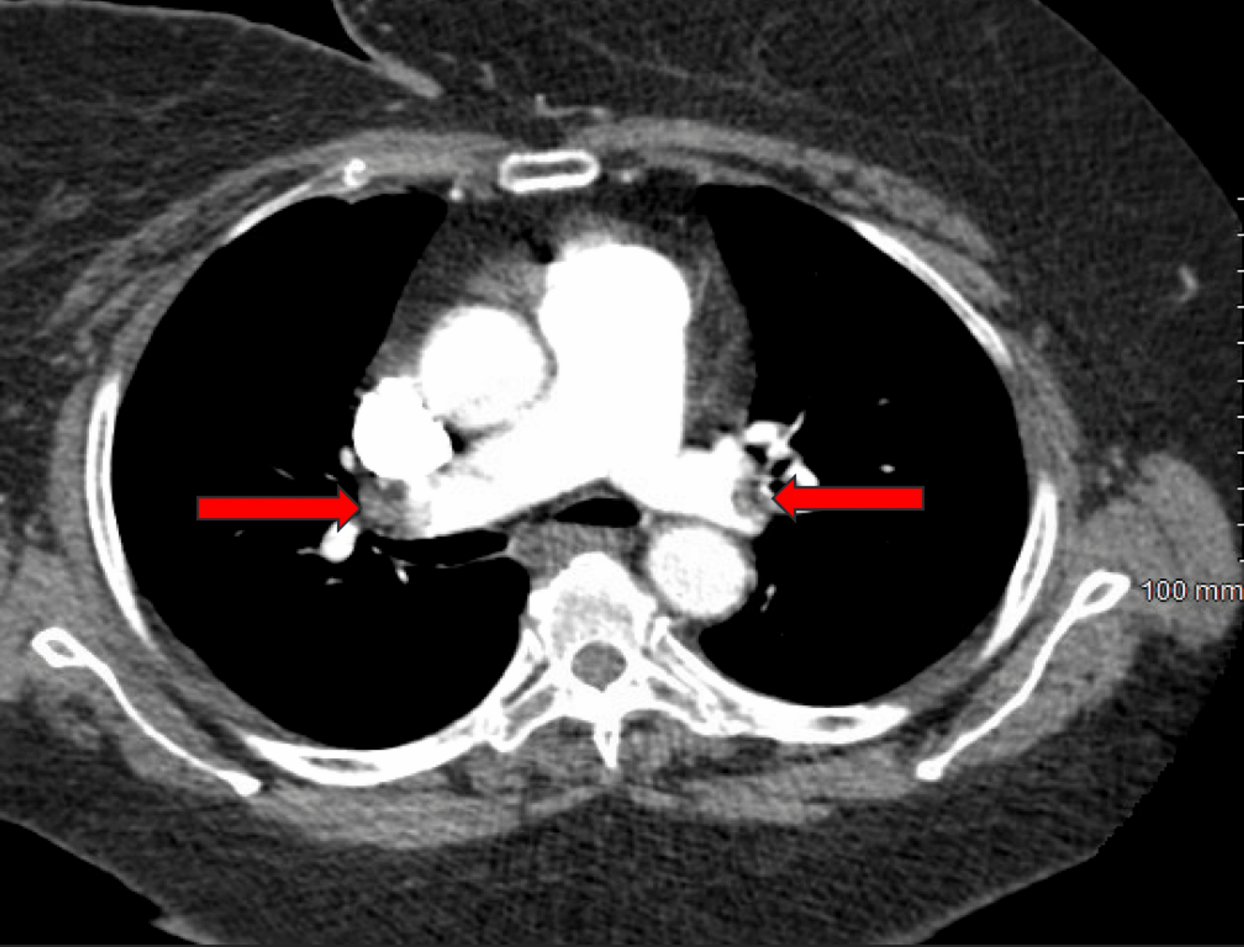
Distal thrombus in both the right and left main pulmonary arteries on CTA of the pulmonary vasculature (arrows). CTA: computed tomography angiography

On day three of hospitalization, he experienced a syncopal episode and had increased oxygen requirements that prompted a computed tomography angiography (CTA) of the pulmonary vasculature (Figure 2). This revealed bilateral, large clot burden pulmonary emboli with distal thrombus in both the right and left main pulmonary arteries.

**Figure 3:**
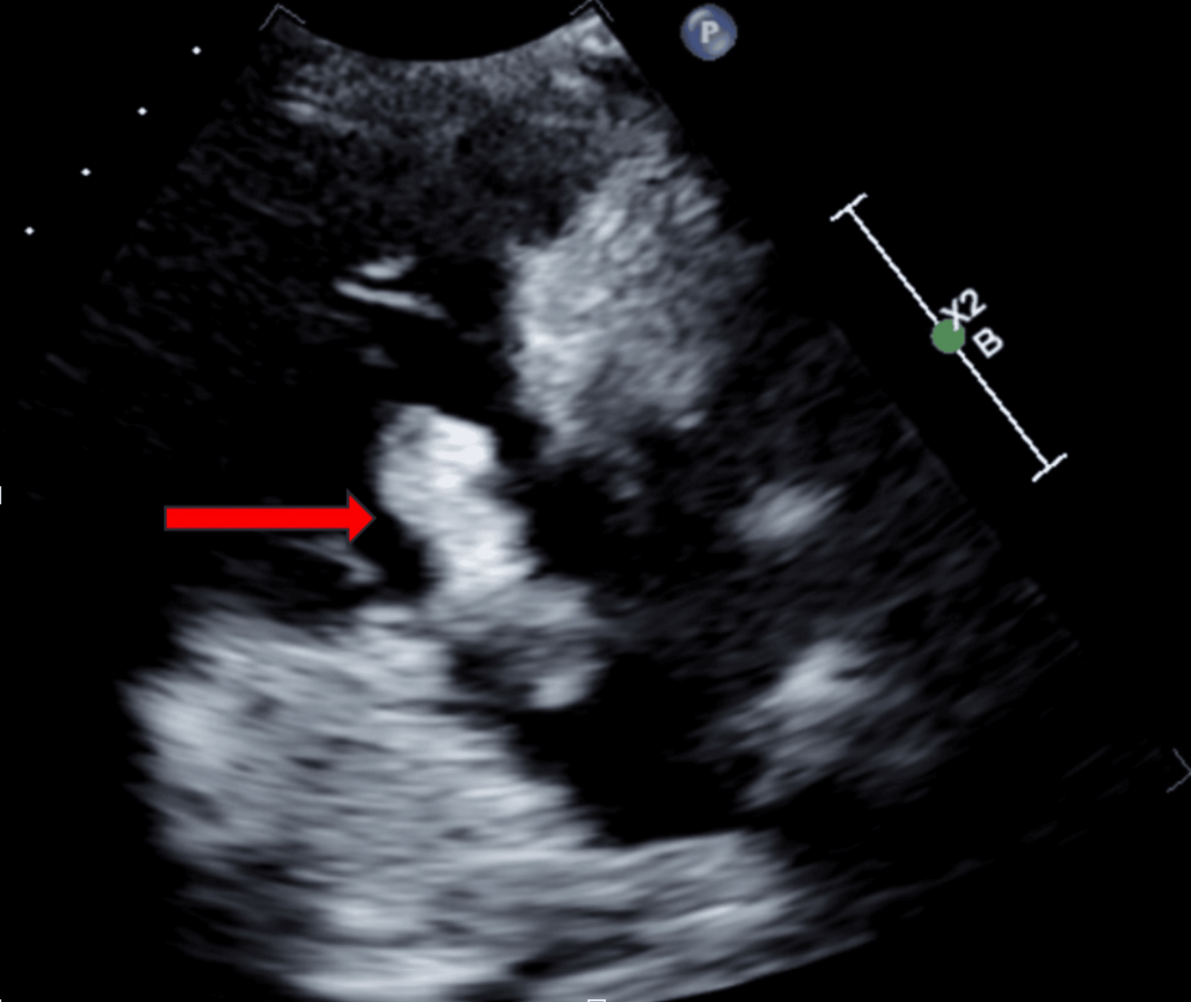
Clot in transit across the tricuspid valve in the right ventricle inflow view (arrow).

Despite the large clot burden, the patient's vital signs remained stable, with a blood pressure of 130/71 mmHg, heart rate of 80 beats per minute, respiratory rate of 18 per minute, and oxygen saturation of 95% on room air, confirming the absence of obstructive shock. Although he was already hospitalized at the time, his calculated PESI and sPESI scores indicated high risk, prompting transfer to the intensive care unit (ICU) for closer monitoring. A transthoracic echocardiogram (TTE) was obtained, and it showed a tethered clot in transit between the right atrium and ventricle, prolapsing across the tricuspid valve (Figure 3).

Cardiology was consulted, and the risks and benefits of various treatment options, including systemic thrombolysis, mechanical embolectomy, and surgical embolectomy, were discussed. Given the life-threatening bleeding risk associated with the invasive measures and risk of in-hospital mortality in addition to his age/frailty and underlying comorbidities, a shared decision was made to proceed with anticoagulation therapy alone, and a do-not-resuscitate order was agreed upon. Therapeutic dose of low-molecular-weight heparin was initiated, but unfortunately, on day eight of hospitalization, he became bradycardic with no recordable blood pressure and went into pulseless electrical activity soon after with an eventual demise.

## Discussion

Pulmonary embolism can be classified as massive or submassive. Newer classifications based on the PESI and sPESI scores, such as high-risk, intermediate high-risk, intermediate low-risk, and low-risk pulmonary embolism, are now used in clinical practice [2-6]. For this case report, we will focus on submassive or intermediate-risk PE. Submassive or intermediate-risk pulmonary embolism is PE associated with right ventricular dysfunction in the absence of hemodynamic instability [2-5]. Right ventricular dysfunction can be assessed based on the findings of a transthoracic echocardiogram (TTE) and computed tomography angiography (CTA) of the pulmonary vasculature, as well as the presence of elevated cardiac markers such as brain natriuretic peptide (BNP) and troponin levels. The presence of these cardiac biomarkers signifies cardiac injury, as they are produced and secreted by the ventricular myocardium into the bloodstream in response to myocardial wall stress. This can be seen in PE, as the increased pulmonary vascular resistance leads to right ventricular strain [7]. Intermediate-risk PE is further classified into intermediate high-risk and intermediate low-risk, which is based on the presence of both or one of the above findings; patients that fall under the intermediate risk require hospitalization as this sub-group is more susceptible to clinical deterioration [3-6].

A right heart thrombus in transit can be defined as a clot that floats between the right chambers of the heart and can include the superior vena cava (SVC) or inferior vena cava (IVC) in some cases. It is commonly associated with a diagnosis of pulmonary embolism with an incidence of 2-6% [2,8,9]. It is a marker for severe PE and can be considered a medical emergency due to its high mortality rate. It is diagnosed by TTE or point-of-care ultrasound (POCUS); Other imaging modalities, such as transesophageal echocardiography (TEE), cardiac magnetic resonance (CMR) imaging, and computed tomography (CT) of the chest, can be used for diagnosis but are rarely required, as TTE is usually adequate [2,8,9]. Right heart thrombi on echocardiography are characterized by their shape, mobility, and size, and can be classified into three main types. Type A thrombi are thin, tubular, and highly mobile, often originating from the lower extremities and representing clots in transit with a high risk of pulmonary embolism; prompt treatment is typically needed. Type B thrombi are ovoid, non-mobile, and broad-based, believed to form in situ due to chamber hypokinesis or dilation; these have a lower embolic risk and are often managed with anticoagulation. Type C thrombi have both mobile and immobile components, combining features of types A and B, and may resemble cardiac tumors, requiring further evaluation to guide management [2,9].

Pulmonary embolism with right heart thrombus presents a complex clinical scenario that may require treatment beyond standard anticoagulation. Systemic thrombolysis is a widely available option that offers rapid administration without requiring specialized equipment or expertise, and it is often considered in hemodynamically unstable patients or when advanced interventions are not accessible [2,9]. Endovascular therapies offer targeted mechanical clot removal and have become increasingly used in select patients. These include the Inari FlowTriever system (Basel, Switzerland: Inari Medical, Inc.), which is FDA-approved for RHT and PE, the AngioVac system (Latham, NY: AngioDynamics, Inc.), the AlphaVac system (Latham, NY: AngioDynamics, Inc.), and the Indigo Aspiration System (Alameda, CA: Penumbra, Inc.) [2,8,9]. Surgical thromboembolectomy remains the most definitive but invasive option, it is reserved for patients who are good surgical candidates and may be preferred in specific anatomic scenarios. Overall, the choice of therapy depends on patient stability, clot characteristics, resource availability, and individual procedural risk [2,8,9]. The adverse effects of the proposed treatment strategies, which include overall morbidity, intracranial hemorrhage, and high bleeding risk, must be taken into consideration [7-11]. While conventional surgical embolectomy remains the most definitive treatment for complex right heart thrombi, it carries substantial risks due to the need for cardiopulmonary bypass (CPB) [2,8,9]. An alternative approach, off-pump surgical thrombectomy, has been proposed in a case reported by Shang et al. This method avoids the use of CPB and was associated with a successful clinical outcome [9]. The off-pump technique may reduce surgical morbidity by eliminating the systemic inflammatory response and coagulation disturbances associated with bypass, but further studies are needed to establish its safety, efficacy, and appropriate patient selection criteria before it can be widely adopted as a standard alternative to traditional surgical embolectomy [10].

In the case presented here, although a right heart thrombus in transit typically signals a high-risk clinical scenario and may prompt consideration of invasive interventions such as systemic thrombolysis, endovascular therapies, or surgical embolectomy, these options were not pursued. This decision was based on the patient's overall clinical frailty and significantly reduced functional reserve.

## Conclusions

In summary, managing pulmonary embolism (PE), particularly when complicated by a right heart clot in transit, remains complex and highly individualized. Anticoagulation therapy is typically the first-line treatment, but the optimal approach for patients with right heart clots continues to be debated. The main options include catheter-based interventions and surgical thrombectomy, with no definitive consensus on which method is superior. The choice of therapy is guided by factors such as the degree of hemodynamic instability, patient comorbidities, and the associated risks and benefits of each intervention.

This case of an 84-year-old male with multiple comorbidities and a mobile right heart thrombus exemplifies the critical importance of individualized decision-making in complex, high-risk clinical scenarios. The patient's advanced age, frailty, and comorbid conditions highlight the importance of a multidisciplinary approach, involving specialists to ensure comprehensive care and informed decision-making. Equally important is patient and family involvement, allowing for shared decision-making that aligns treatment with the patient's preferences and goals. The fatal outcome emphasizes the need for thorough risk-benefit evaluation and underscores the value of team-based care and shared decision-making in optimizing patient outcomes. Further studies and registries are necessary to guide treatment strategies for such complex cases and provide more precise guidance for clinicians navigating these high-risk clinical situations.
